# Urinary catheter-associated microbiota change in accordance with treatment and infection status

**DOI:** 10.1371/journal.pone.0177633

**Published:** 2017-06-19

**Authors:** Laetitia Bossa, Kimberly Kline, Diane McDougald, Bonsan Bonne Lee, Scott A. Rice

**Affiliations:** 1 Neuroscience Research Australia (NEURA), Sydney, New South Wales, Australia; 2 The Singapore Centre for Environmental Life Sciences Engineering, The School of Biological Sciences, Nanyang Technological University, Singapore; 3 The ithree Institute, The University of Technology Sydney, Sydney NSW Australia; 4 Prince of Wales Hospital, Spinal Medicine Department, Randwick, NSW, Australia; 5 The Centre for Marine Bio-Innovation, The School of Biological, Earth and Environmental Sciences, The University of New South Wales, Sydney, NSW Australia; University of Illinois at Urbana-Champaign, UNITED STATES

## Abstract

The use of long-term catheterisation to manage insensate bladders, often associated with spinal cord injury (SCI), increases the risk of microbial colonisation and infection of the urinary tract. Urinary tract infection (UTI) is typically diagnosed and treated based on the culturing of organisms from the urine, although this approach overlooks low titer, slow growing and non-traditional pathogens. Here, we present an investigation of the urinary tract microbiome in catheterised SCI individuals, using T-RFLP and metagenomic sequencing of the microbial community. We monitored three neurogenic patients over a period of 12 months, who were part of a larger study investigating the efficacy of probiotics in controlling UTIs, to determine how their urinary tract microbial community composition changed over time and in relation to probiotic treatment regimens. Bacterial biofilms adherent to urinary catheters were examined as a proxy for bladder microbes. The microbial community composition of the urinary tract differed significantly between individuals. Probiotic therapy resulted in a significant change in the microbial community associated with the catheters. The community also changed as a consequence of UTI and this shift in community composition preceded the clinical diagnosis of infection. Changes in the microbiota due to probiotic treatment or infection were transient, resolving to microbial communities similar to their pre-treatment communities, suggesting that the native community was highly resilient. Based on these results, we propose that monitoring a patient’s microbial community can be used to track the health of chronically catheterized patients and thus, can be used as part of a health-status monitoring program.

## Introduction

Catheterised patients, particularly those with spinal cord injuries (SCI), are highly susceptible to microbial colonization and hence are at increased risk of symptomatic infection [1–8]. SCIs are often associated with neurogenic bladders, a condition in which nerve damage gives rise to bladder dysfunction, necessitating catheterization. Catheters can facilitate biofilm formation on their surfaces and this can subsequently provide direct access to the bladder, leading to colonization. These biofilms serve as reservoirs for infection by a variety of organisms [8–12]. Indeed, the isolation of multiple organisms at high titers from the urine is not uncommon for catheterised patients [3,13–16]. Increasingly, it is appreciated that urinary tract infections may involve non-traditional pathogens or may be caused by multiple infecting organisms [17]. It may therefore be possible to use molecular approaches to improve our understanding of the roles of different microorganisms and their roles in symptomatic and asymptomic colonization or infection of catheterised patients to better enable strategies to mitigate symptomatic infections.

DNA fingerprinting techniques such as T-RFLP have proven to be useful in characterising microbes in soil and marine environments [18,19], and to a lesser extent in humans. For example, Wang et al. (2004) monitored the faecal microbiome of two infants during a longitudinal study using T-RFLP of the 16S rRNA gene and DNA sequencing and found a shift in the community after weaning compared to a stable composition during breastfeeding [20]. Changes in the urine microbiomes of renal transplant patients were linked to transplant rejection and infection episodes, suggesting that such changes in community composition can be predictive of poor outcomes for patients [21]. For example, a 16S rDNA based analysis of urinary catheters of non-neurogenic bladders in males showed that the communities varied significantly between patients [22]. Similar findings were reported in another study of the microbiota of catheters of patients with neurogenic bladders and additionally reported, that the specific organisms identified depended on the method used, e.g. T-RFLP or pyrosequencing [23]. The interpatient variation in microbiome members also holds true for neurogenic and non-neurogenic bladders, and often includes organisms that have not previously been described by culturing [24]. What remains to be understood is how those communities change over time, especially in relation to clinically relevant infections as well as antibiotic and alternative therapies.

With the rise of multidrug and extremely drug resistant organisms, there is a dire need for alternative strategies to control UTI, especially for highly at risk patients, such as spinal cord injury patients. One strategy that has gained attention is the use of probiotics as a type of bioregulation or biointerference against UTI. *Lactobacilli* spp. and *Bifidobacteria* spp. are the most commonly investigated probiotics for altering the microflora and preventing disease in the human host [25,26] and their efficacy is currently being tested for their ability to reduce UTI associated with neurogenic bladders [27]. Administration of *Lactobacillus rhamnosus* GR-1 and *Lactobacillus reuteri* RC-14 orally and intravaginally can alter the composition of the microflora at intestinal and urogenital sites [28,29]. In vitro studies have shown that catheters coated with *Escherichia coli* 83972 resist colonisation by pathogenic *Enterococcus faecalis*, *Providencia stuartii*, lactose negative *E*. *coli* and *Candida albicans* [30,31]. Direct instillation of *E*. *coli* 83972 into the neurogenic bladder is safe and results in successful, but transient, colonisation [32] and treated subjects experienced fewer incidences of UTI [32,33]. One of the primary challenges in using *E*. *coli* 83972 as a long term therapeutic was its lack of persistence in the bladders of some patients [32,33]. This highlights the need to monitor and understand changes in the urogenital microbiome as a function of probiotic treatment as well as the long-term effects of the probiotic once treatment has stopped.

Here, we present data from a clinical study on the efficacy of probiotic treatments for the control of UTIs in neurogenic patients in which we assessed the impact of probiotics on the host urinary tract flora. Three patients were monitored longitudinally for changes in their urinary microbiome both during and after the probiotic treatment period. Bacteria were identified and classified by DNA fingerprinting and next generation amplicon sequencing. The results show that the microbiomes changed in response to treatment and UTI status and suggests that such data can be used to monitor patients’ health status and can be used to tailor management practices for individuals.

## Materials and methods

### Ethics statement

Patients were participants in the ProSCIUTTU Clinical Trial, Australian New Zealand Clinical Trials Registry number ACTRN 12610000512022. All study protocols were approved by the relevant Human Research Ethics Committees at Prince of Wales Hospital, Royal North Shore Hospital and Royal Rehab (HREC ref no. 11/036, HREC/10/HARBR/102; SSA/10/HAWKE/171). Written informed consent was obtained from all participants and samples were de-identified by assigning them an arbitrary subject number.

### ProSCIUTTU clinical trial treatment protocol

Inclusion and exclusion criteria for patient enrolment were as described in the ProSCIUTTU trial protocol [34]. Three patients from the trial were approached and provided informed consent for the longitudinal study of their catheter flora. The longitudinal design allowed for the study of catheter flora during the ProSCIUTTU probiotic treatment period and afterward during no treatment. Subjects were selected by convenience sampling on the following criteria: completion of ProSCIUTTU enrolment before April 2013, allowing sufficient time for collection and analysis of post-treatment samples, and ease of access. Patients 2 and 3 resided in close proximity to Prince of Wales Hospital, which simplified the logistics of sample collection.

While enrolled in ProSCIUTTU, patients underwent 6 months of oral treatment with probiotic formulations consisting of *Lactobacillus rhamnosus* GR-1 with *Lactobacillus reuteri* RC-14 or placebo; and *Lactobacillus rhamnosus* GG (LGG) with *Bifidobacterium* (BB-12) (CHR Hansen, Denmark) or placebo, as per the randomisation protocol [34].

Patient 1 received GR1-RC14 active and LGG-BB12 placebo. Patients 2 and 3 received the GR1-RC14 placebo and LGG-BB12 active. Subjects and investigators were blinded to treatment allocation during enrolment. UTI history was collected for the year prior to ProSCIUTTU enrolment and patients were contacted fortnightly to monitor UTI status, compliance with study protocol and changes in medication.

### Catheter sample collection

Between the time points of Month 0 (Baseline) and Month 6 of enrolment, an explanted urinary catheter sample was collected from each patient. In addition, samples were collected from each patient at multiple time points in the post-treatment period, with a goal of six consecutive samples. The collection of the explanted catheter coincided with the patient’s regular catheter change and was performed aseptically by trained health personnel. A 5 cm section of the bladder end of the catheter was cut using sterile scissors and collected in a sterile container. All samples were labelled with a patient ID, date and time removed. The catheter was stored at 4°C, transported laboratory and the adherent community was extracted within 48 h of collection. This process was previously shown to have no effect on quantitative counts of microorganisms [35–38] and hence should not introduce a bias in the data.

### Collection of and DNA extraction from catheter biofilms

Under sterile conditions, the catheter was transferred to a falcon tube containing 5 ml of sterile phosphate buffered saline (PBS) and sonicated in an ultrasonic water bath (Unisonics, Australia) at 90 kHz for 5 min to dislodge adherent microorganisms. This was followed by vortexing (Whirli VIB 2, Inter Med, Denmark) for 90 s.

After removal of the catheter from the falcon tube, the cell suspension was centrifuged at 7690 *g* and 4°C for 10 min (Universal 320R, Hettich, Germany), the supernatant was discarded and the biomass was stored at -20°C. Total DNA was extracted using the FastDNA SPIN Kit for Soil and the FastPrep Instrument (MP Biomedicals, Santa Ana, CA) according to manufacturer’s instructions. The quality of DNA was confirmed by electrophoresis on a 1% (w/v) agarose gel stained with ethidium bromide and the concentration and purity determined by spectrophotometery (Nanodrop 1000; Thermo Fisher Scientific, MA, USA).

### Biofilm/microbial community analysis by T-RFLP

#### Polymerase Chain Reaction (PCR)

The primers 27F (5'-AGR GTT TGA TCM TGG CTC AG-3') and 536R (5’-GWA TTA CCG CGG CKG CTG-3’) were used for amplification of bacterial 16S rRNA genes. The 5’ end of the 27F primer was fluorescently labeled with 6-carboxyfluorescein (6-FAM). The PCR reaction mixture contained: 25 μL EconoTaq PLUS mastermix (0.1 units/μL EconoTaq DNA polymerase, Reaction Buffer (pH 9.0), 400 μM dNTP, 3 mM MgCl_2_ and PCR Enhancer/Stabilizer (Lucigen, MI, USA); 10 μM each of forward and reverse primers; 50–100 ng of template DNA and ultrapure water up to 50 μL.

The PCR reactions were performed in a MultiGene Gradient Thermal Cycler (Labnet International Inc., NJ, USA) with the following parameters: initial denaturation at 95°C for 2 min followed by 30 cycles of denaturation at 95°C for 30 s, annealing at 55°C for 30 s and extension at 72°C for 1 min. The final extension step was at 72°C for 10 min. PCR products were purified using a PureLink PCR Purification Kit (Life Technologies, MA, USA) according to the manufacturer’s instructions. Following amplification and purification, the quality of amplified DNA was confirmed by spectrophotometry (Nanodrop; Thermo Fisher Scientific, MA, USA).

#### Restriction endonuclease digestion

The fluorescently labelled amplicons were digested using *Msp*I (New England Biolabs, MA, USA), purified using an Oligo Clean and Concentrator Kit (Zymo Research Corporation, CA, USA), diluted to a concentration of 2 ng/μL and submitted to the Ramaciotti Centre for Genomics (University of New South Wales, Sydney, Australia) where the fragments were separated by size on a 3730xl capillary sequencer (Life Technologies, NY, USA).

#### T-RFLP data analysis

Raw data files consisting of peak size and intensity were tabulated and electropherograms were plotted using Peak Scanner Software (Applied Biosystems, CA, USA). Peak sizes were determined by comparison to the size standard GS600LIZ with a peak detection range for electropherograms between 30 to 600 bp. The minimum peak height threshold for baseline detection of peaks was 50 fluorescent units. Samples that were indicated by Peak Scanner software to be of poor quality were discarded (if < 75% of size standard fragments were detected). Peak data tables were exported into Excel, reformatted and then imported into T-RFLP Analysis Expedited Software (T-REX) [39] for noise filtering and alignment of peaks between samples.

In T-REX, the noise filtration algorithm used peak area with a standard deviation multiplier of 3. The peak alignment algorithm rounded off-peak sizes to the nearest integer and clustered peaks with a threshold of 0.5. The resultant two-way data matrix of T-RFLPs by samples was imported into multivariate statistical software package Primer 6 (Primer-E Ltd, Plymouth, UK). The relative abundance of T-RFLPs was fourth root transformed to reduce the impact of the more abundant T-RFLPs and to prevent understating the importance of the less abundant T-RFLPs. Bray Curtis similarity coefficients were calculated for each pair of samples creating a Bray Curtis similarity matrix. Bray Curtis similarity between samples is based on the relative abundances of different taxa in the given samples. The similarity data was graphed as two-dimensional, non-parametric multi-dimensional scaling (MDS) plots for visual exploratory analysis of differences between samples. Permutational multivariate analysis of variance (PERMANOVA) was used to analyse inter- and intra-patient differences in microbial communities.

### Microbial community analysis by Illumina sequencing

The microbial community was identified by 16S rRNA gene sequencing of the whole community using the Illumina sequencing platform. The primers used for amplification of bacterial 16S rRNA genes were modified 27F and 536R primers with Illumina adapters attached to the 5’ ends as follows:

Forward Primer: 5’ TCGTCGGCAGCGTCAGATGTGTATAAGAGACAGAGA GTTTGATCMTGGCTCAG

Reverse Primer: 5’ GTCTCGTGGGCTCGGAGATGTGTATAAGAGACAGGWA TTACCGCGGCKGCTG

Amplicons were verified by electrophoresis on a 1% agarose gel. PCR amplicons were purified with Agencourt AMPure XP beads (Beckman Coulter Inc., CA, USA). PCR products were purified by the addition of 20 **μ**L of AMPure XP beads (Beckman) to the amplicons. Purified PCR amplicons were submitted to the Singapore Centre for Environmental Life Sciences Engineering at Nanyang Technological University, Singapore for processing. Sequencing was performed on a single plate on the Illumina GAIIx platform.

#### Illumina sequencing data processing

Data was cleaned and analysed using the Quantitative Insights Into Microbial Ecology (QIIME) software package and pipeline [40]. Quality trimming was done using Cutadapt v1.8, where only truncated reads with a quality score of 20 and length of at least 50 base pairs were kept for downstream analysis. Reads were 300 bp in length and the minimum quality score required for each base call in a read was 20. Reads were truncated where two or more consecutive base calls registered a quality score below 20. Additionally, the algorithm discarded reads with ambiguous base character N in their sequence. Following quality filtration, there were 11,969,697 paired-end Miseq reads which were suitable for downstream analysis. The forward and reverse reads were merged into a single read using FLASH v1.2.1, with minimum overlap of 10 bp and maximum of 300 bp. Paired-end reads that did not merge were discarded. The sequences have been submitted to Genbank under the Bioproject ID: PRJNA389364.

#### Operational Taxonomic Unit (OTU) analysis

Filtered reads were de novo clustered into OTUs by 97% similarity. The most abundant read in the OTU group was selected as the representative sequence and taxonomy was assigned using Greengenes as the reference database [41,42].

#### Taxonomic assignment

Chimeras were removed prior to clustering and alignment using the chimera slayer [43]. Taxonomy was assigned to sequences using UCLUST algorithm against Greengenes as the reference database [41,42]. Phylogenetic beta-diversity was calculated using weighted and unweighted UniFrac distances between samples in QIIME [44].

The OTU table of sequencing data containing 270 genera and their abundances for each sample created in QIIME was imported into Primer 6 software (Primer-E Ltd, Plymouth, UK) for visual exploratory analysis of the similarity between samples and for hypothesis testing. The data matrix of T-RFLP peak size and intensity was also imported into Primer. Taxonomic profiles were created for each sample from abundance data from both sequencing and T-RFLP. Visual analysis was done using non-metric, multi-dimensional scaling (MDS) plots. MDS plots were generated from the ranked Bray Curtis distances between samples. In addition to MDS plots, Principal Coordinates Ordination (PCO) was used to visualise similarity/differences between samples. For sequencing data, weighted UniFrac distances, calculated based on phylogenetic differences between OTUs, were used to assess associations between Patient ID and community composition. Three-dimensional Principal Coordinates of Analysis PCoA plots were generated from the weighted UniFrac distance matrix of Illumina sequencing data. Hypothesis testing for effects of Patient ID and probiotic therapy on flora composition was done by Permutational Multivariate Analysis of Variance, PERMANOVA [45]. Each factor (Patient ID and probiotic therapy) was tested separately using single factor PERMANOVA analysis with unrestricted permutation of raw data and Type III (partial) sums of squares. Hypothesis testing for differences in microbial diversity was done using students t-tests. A significant difference was accepted for p values of 0.05 or less.

## Results

### Patient demographics and clinical data

A randomised, controlled clinical trial (ProSCIUTTU) was conducted in Australia in 2011–2014 examining the use of probiotics in the prevention of UTI in the spinal cord injured population [27]. Three patients that had completed ProSCIUTTU enrolment before April 2013 allowing sufficient time for collection and analysis of post-treatment samples, were selected for analysis of their microbiomes (Table 1). Patient 1 had the longest duration of neurogenic bladder while Patient 3 had a neurogenic bladder for less than one year. Patients 1 and 3 had suprapubic catheters while Patient 2 had an indwelling urethral catheter.

**Table 1:** Patient characteristics.

Patient	Age (years)	Gender	Injury level and completeness ^a^	Duration of neurogenic bladder	Catheter type ^b^
1	50	Male	C3 ASIA A	11 years	Suprapubic
2	62	Male	C5/C6 ASIA B	8 years	Indwelling urethral
3	53	Male	C4 ASIA B	< 1 year	Suprapubic

^a^ ASIA, American Spinal Injury Association Impairment Score

^b^ Subprapubic catheters are inserted via an incision through the abdomen; indwelling catheters are inserted via the urethra.

Twenty five samples were analysed, where nine of these were from Patient 1. For this patient, samples collected during the probiotic treatment were at Months 1, 3 and 6. During the post-treatment period, an additional 6 samples were collected. Post-treatment sample collection lasted for a period of nine months, commencing 15 months after completion of the probiotic trial. The average duration of implantation for each catheter for Patient 1 was 57.8 days with a standard deviation of 27.7 days. The variation in duration of implantation times was due to the development of catheter blockage from crystal formation. For example, post-treatment sample 4 corresponded to the emergency change of a blocked catheter, 15 days after insertion. Patient 1 was asymptomatic and experienced no episodes of clinically significant UTI throughout the entire study.

Seven samples were collected from Patient 2, where only one sample, Month 6, was collected during the probiotic interventional period. Months 0 (pre-treatment) and 3 were not collected and six samples were collected in the post-treatment period. The first post-treatment sample was retrieved 22 months after completion of the probiotic trial, and follow-up continued over a six-month period. The average duration of implantation for Patient 2 catheters was 25.1 days with a standard deviation of 10 days. The patient suffered from one clinically significant UTI during follow-up, most likely *E*. *coli* based on urine culture analysis, resulting in an emergency catheter change but the sample was not collected for the study. As a consequence of the infection, the patient received antibiotic treatment for the UTI.

Patient 3 supplied nine samples, collected at Months 0 (pre-treatment), and Months 3, 5 and 6 during probiotic treatment. Five samples were collected after completion of the probiotic trial, and sample collection for the post-treatment period commenced 1 month after the clinical trial and lasted six months. Catheters for Patient 3 were implanted for an average duration of 31 days with a standard deviation of 4.9 days. The patient experienced no clinically significant UTI episodes during the ProSCIUTTU study or in the post-treatment follow-up.

### Microbiome composition by patient

**Fig 1:**
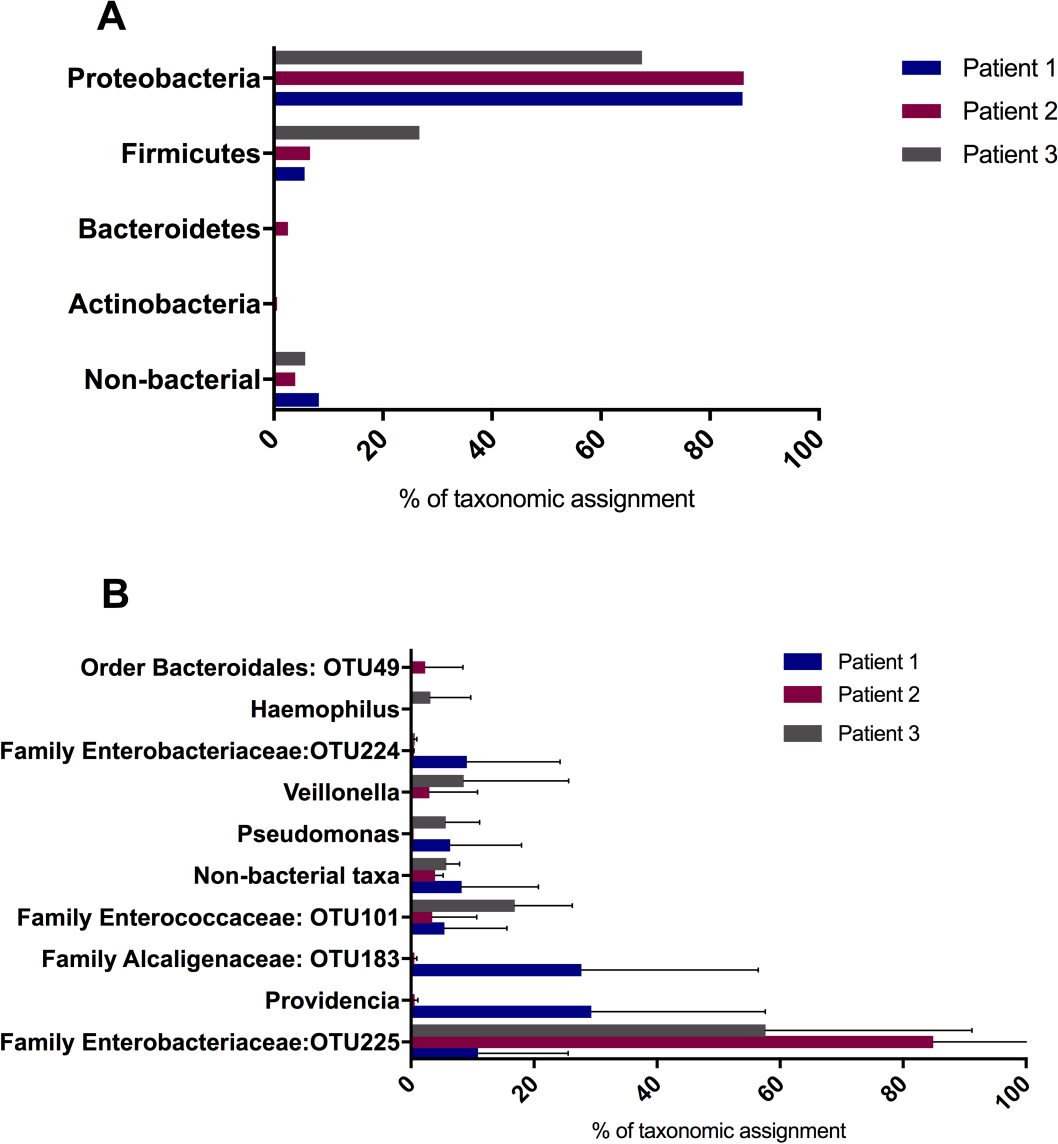
Taxonomic distribution of microorganisms in catheterized patients. **(**A) Phyla as a percentage of taxonomic assignment in urinary catheter microbiota of the three patients across probiotic treatment and post-treatment time points based on meta-community sequence analysis. (B) Genera as a percentage of taxonomic assignment for the three patients across probiotic treatment and post-treatment time points based on meta-community sequence analysis. Only genera with an average representation of 1% or greater are shown. Error bars show standard deviations. OTUs that were not resolved at the genus level are referred to by their lowest identified phylogenetic classification and OTU number. Only phyla with a representation of at least 1% in a single individual’s catheter microbiome are shown.

Amplicon based sequencing was used to identify and quantify urinary catheter associated microbial community members and sequences were grouped together as OTUs and reported when those organisms represented at least 1% of the total community. When the microbiomes were analysed, Patient 1 was colonised by 10 OTUs, and Patients 2 and 3 had 9 OTUs each. Across the three patients, *Proteobacteria* was the dominant phylum (Fig 1A), averaging 79% of the total bacterial community sequences. *Firmicutes* were the second most abundant phyla, with a mean representation of 13%. Across all three patients, *Bacteroidetes* and *Actinobacteria* represented an average of less than 1% each while non-bacterial sequences (e.g. fungal and human sequences etc) made up an average of 6% of sequences. Patient 1 had the highest average percentage of non-bacterial taxa at 8%, followed by Patient 2 at 4% and Patient 3 at 6%.

The community members were then classified to genus level. Where the assignment was not clear at the genus level, the assignment was given as an unclassified genus within the relevant family and are referred to by their OTU designator. The most abundant OTU belonged to an unclassified genus of the family *Enterobacteriaceae*/OTU225 (average of 48%), followed by *Providencia* (11%), the unclassified genus of the family *Alcaligenaceae* (10%), and an unclassified genus of family *Enterococcaceae*/OTU101 (9%) (Fig 1B). Non-bacterial taxa represented 6% of sequences. Other abundant genera were *Pseudomonas* and *Veillonella*, each making up an average of 4% of the total community across the three patients. Sequences of the family *Enterobacteriaceae*/OTU224, genus *Haemophilus* and order *Bacteriodales*/OTU49 each represented on average between 1 and 3% of the total community.

The two taxa that were represented at 1% or more in the flora across all three patients were the unclassified genus of the family *Enterobacteriaceae* (OTU225) and the unclassified genus of the family *Enterococcaceae* (OTU101). The percentage of *Enterococcaceae* was highest in Patient 3 (17%), then Patient 1 (5%) and was lowest in Patient 2 (3%). Patient 2 had the highest representation of *Enterobacteriaceae* (85%), followed by Patient 3 (58%) and then Patient 1 (11%).

### Inter-patient variation in microbial communities

**Fig 2:**
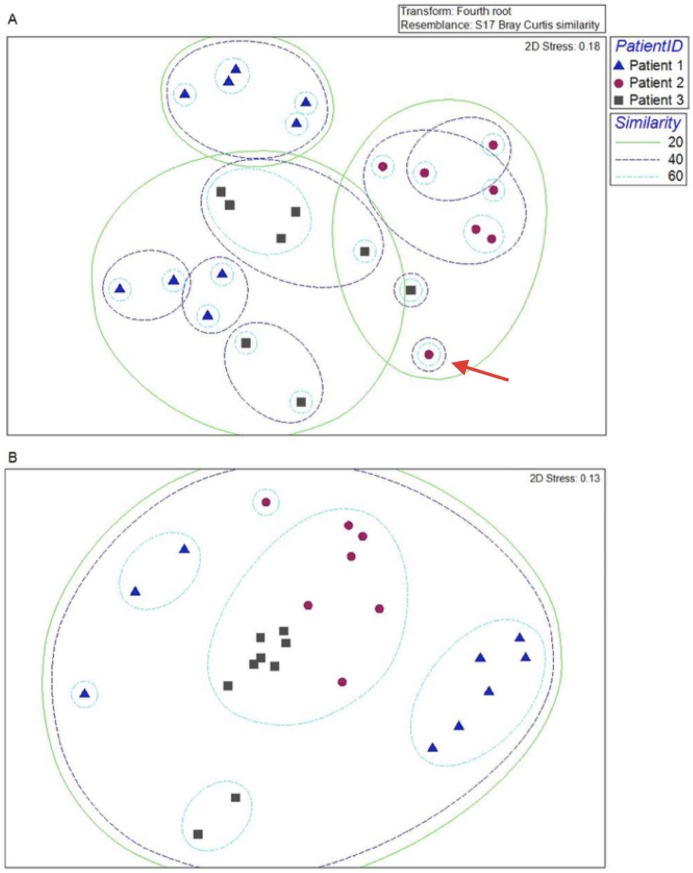
MDS plot showing inter-patient differences in microbial communities of the three patients at time points of probiotic treatment and post-treatment. Data are shown for T-RFLP analysis (A) and metacommunity sequence analysis (B). The microbial flora for Patient 1 (Blue triangles), Patient 2 (Red circles) and Patient 3 (Grey squares) were plotted for each time point collected. The red arrow indicates the pre-UTI sample for patient 2. The contours group samples with a given percentage of similarity (20%, 40% and 60%) based on composition and relative abundances of taxa present.

To examine differences in urinary tract microbiota between patients, T-RFLP and sequencing data were compared. Examination of the T-RFLP data (Fig 2A) showed that the samples clustered together by patient. For example, all of Patient 2’s samples clustered into a single group (40% similarity based on Bray-Curtis analysis, red circles), except for the pre-UTI sample (red circles, arrow) (Fig 2A). Similarly, the samples from Patients 1 and 3 (blue triangles and grey squares, respectively) formed separate clusters, and these clusters were distinct for each individual. The separation of the samples for patients 1 and 3 into two clusters was associated with whether the patients were on or off of the probiotic treatment (described in detail below). This clustering suggests that the communities were significantly different between patients, with a high intrapatient similarity (PERMANOVA, p = 0.001). Community sequencing data gave similar results as the T-RFLP data (Fig 2B), which also indicated that the microflora of the three patients had significant inter-patient differences. Within patient data were similar with two distinct clusters that correlated with probiotic treatment (PERMANOVA, p = 0.001), with pre-treatment and post-treatment microflora being similar. While there was a trend of patients having distinct microbiomes, based on the community sequencing data, Patients 2 and 3 were more similar to each other than to Patient 1, which was the most different from the other two patients.

**Fig 3:**
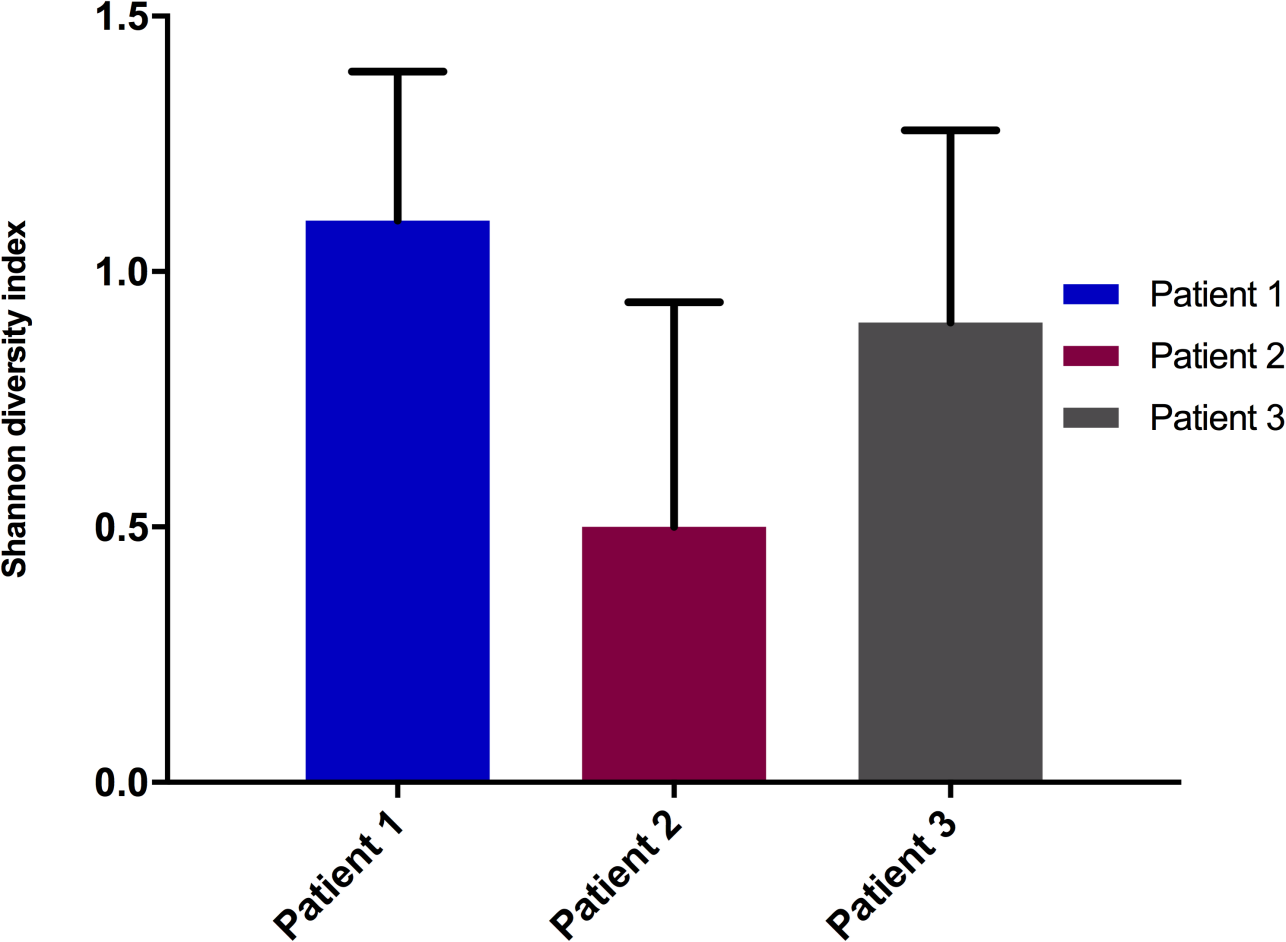
Shannon diversity indices of the three patients at the genus level from meta-sequencing data.

To compare the communities between patients, Shannon-Wiener diversity indices were calculated for the microbial community of each patient based on the Illumina sequence data (Fig 3). Single factor ANOVA showed that the microbial diversities of the three patients were significantly different (F of 5.9 > F_crit_ of 3.4). Based on two-tailed student’s t-tests, the community diversity indices for Patients 1 and 3 were not significantly different (t Stat 0.98 < t Critical two-tail 2.13). In contrast, the diversity of the microbiota for Patient 2 was significantly different from that of Patient 1 (t Stat 3.22 > t Critical two-tail 2.23) and was also significantly different from Patient 3 (t Stat -2.22 < -t Critical two-tail 2.18). Overall, Patient 2 had lower microbial diversity, but higher variance, compared to Patients 1 and 3. Interestingly, Patient 2 had a history of recurrent symptomatic UTI, while Patients 1 and 3 did not. It should also be noted that Patient 2 had an indwelling urethral catheter, while Patients 1 and 3 had suprapubic catheters.

### Changes in microbial community composition during vs after pro-biotic intervention

As indicated above, the microbial communities of each patient tended to form two distinct clusters that were differentiated by probiotic treatment. Therefore, to determine if a patient’s microbiome differed during probiotic therapy compared to post-probiotic treatment, samples of each patient were examined across time points using both T-RFLP and sequencing approaches. The results were compared using MDS plots and Principal Coordinates plots and PERMANOVA was used to test for statistical significance.

**Fig 4:**
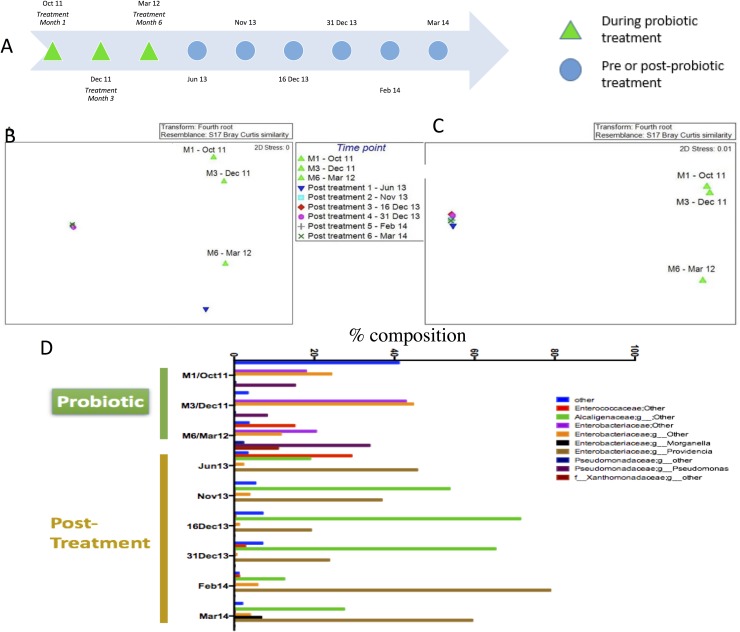
The microbial community composition from Patient 1 during and after probiotic treatment. (A) Samples and time line for Patient 1. MDS plot of the microbial flora from Patient 1 based on T-RFLP (B) and Illumina sequencing data (C). The green triangles represent samples collected from October 2011 to March 2012 corresponding to Months 1, 3 and 6 of probiotic treatment. All other samples were collected at six time points post-treatment, between June 2013 and March 2014. (D) Microbiome composition at the genus level for Patient 1 during and after probiotic treatment based on Illumina sequencing data. The three samples on the left were collected from October 2011 to March 2012 corresponding to Months 1, 3 and 6 of probiotic treatment. The other samples were collected at six time points in the post-treatment period, between June 2013 and March 2014. Only OTUs representing at least 1% of the total community are shown. OTUs that were not resolved at the genus level are referred to by their lowest identified phylogenetic classification.

For Patient 1, three samples were collected during the probiotic treatment at Months 1, 3 and 6 respectively (green triangles). Six samples were collected from the post-treatment follow-up (non-green triangles). MDS plots showed temporal differences between probiotic (green triangles) and post-probiotic samples (non-green triangles). Comparison of the communities by T-RFLP and by sequence analysis by MDS plot (Fig 4A and 4B) both indicated that there was a significant difference between the communities during and post probiotic intervention (p = 0.022 for T-FRLP and p = 0.012 for sequencing data, PERMANOVA). This was especially clear for the 16S sequence data where the communities segregated into two distinct groups based on treatment (green triangles on the right in Fig 4B) or no treatment (samples on the left in Fig 4B).

To further understand the differences in communities between the samples collected at different time points, the community sequence data were examined based on changes in OTUs at the genus level for each time point. Based on OTUs that were present at >1% of the total community, 9 different bacterial OTUs were identified at the genus level during the probiotic treatment (October 2011 to March 2012) and the follow-up periods (June 2013 to March 2014) (Fig 4C). During probiotic treatment, the dominant taxa for Patient 1 were OTUs 224 and 225, both of which correspond to the family *Enterobacteriaceae* at an average of approximately 27% each and OTU254 of genus *Pseudomonas* at an average of 19% (Fig 4). When probiotic treatment was stopped, the microbiomes changed and were dominated by OTU233 of genus *Providencia* at 44% and OTU183 of family *Alcaligenaceae* at 42%, both of which were undetectable during the probiotic treatment period (Fig 4C). Additionally, non-bacterial taxa declined from an average of 16% during probiotic treatment to 4% when treatment was stopped.

**Fig 5:**
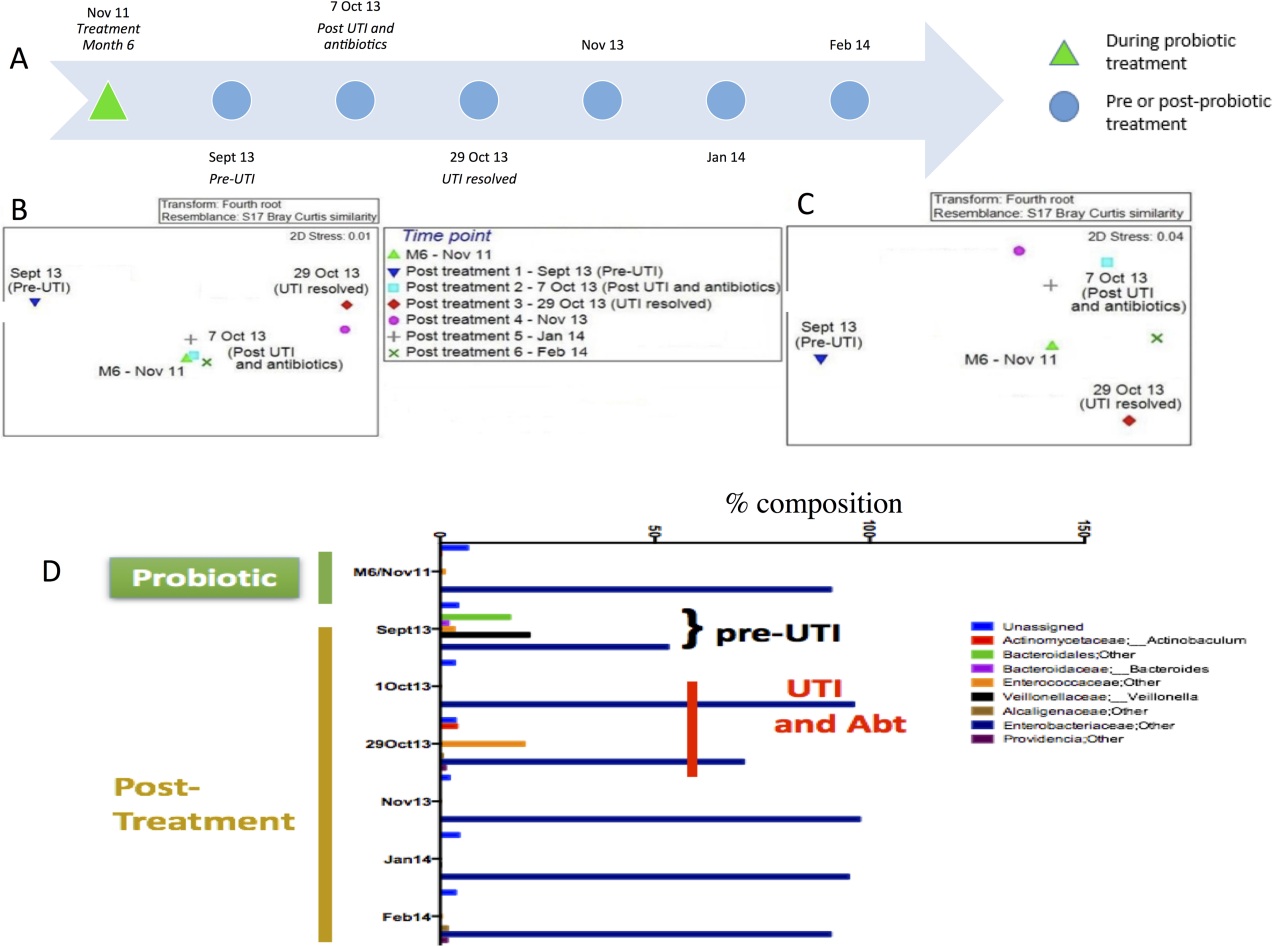
The microbial community composition from Patient 2 during and after probiotic treatment. (A) Samples and time line for Patient 2. MDS plot of the microbial flora from Patient 2 during and after probiotic treatment based on T-RFLP (B) and Illumina sequencing data (C). The green triangle represents the sample collected in November 2011 at Month 6 of probiotic treatment. All other samples were collected post-treatment between September 2013 and February 2014. The plot also shows time points prior to and after UTI occurrence and antibiotic treatment between September and October 2013. D. Microbial community composition at the genus level for Patient 2 during and after probiotic treatment, and prior to and after UTI occurrence based on Illumina sequencing data. The sample on the left was collected in November 2011 corresponding to Month 6 of probiotic treatment. The other samples were collected at six time points in the post-treatment period, between September 2013 and February 2014. The September 2013 sample was at a time point prior to UTI. Only OTUs representing at least 1% of the total community are shown. OTUs that were not resolved at the genus level are referred to by their lowest identified phylogenetic classification.

For Patient 2, there was only one sample collected during probiotic treatment (green triangle) and this was at Month 6. Six samples were collected from the post-treatment follow-up (non-green triangles). MDS plots showed no spatial separation of the probiotic sample represented by the green triangle, from the post-probiotic samples (Fig 5A and 5B). However, there was spatial separation of one sample from the others, represented by the inverted blue triangles in Fig 5A and 5B. This sample was collected in September 2013, prior to a clinically significant UTI, which occurred in early October 2013. This pre-UTI sample segregates from samples collected at non-UTI time points, suggesting that there was a disturbance in the microbial community associated with the onset of symptomatic infection.

Community sequence data was examined for further investigation of the differences in communities between the samples collected at different time points, in particular changes in types and relative abundances of OTUs at the genus level. Based on a sub-analysis of the OTUs that were present at >1% of the total community for patient 2, eight different bacterial genera over the probiotic treatment period (based on Month 6 sample of Nov 2011) and the follow-up periods (September 2013 to February 2014) as shown in Fig 5C. The dominant taxon was OTU 225 from the family *Enterobacteriaceae*, representing an average of 85% of the total community. Whilst *Enterobacteriaceae* was dominant at all time points, it decreased to 53% in the pre-UTI sample of September 2013 (Fig 5C). The microbial community of the pre-UTI sample also showed increased diversity of OTUs, and was the only sample with the presence of *Veillonella*, representing 21% of the community, and order *Bacteroidales* at 16%. The pre-UTI community also has 3% of the family *Enterococcaceae* and 2% of phylum *Bacteriodes*. Thus, in contrast to patients 1 and 3, patient 2 appeared to have a much higher proportion of Bacteriodetes and Actinobacteria.

**Fig 6:**
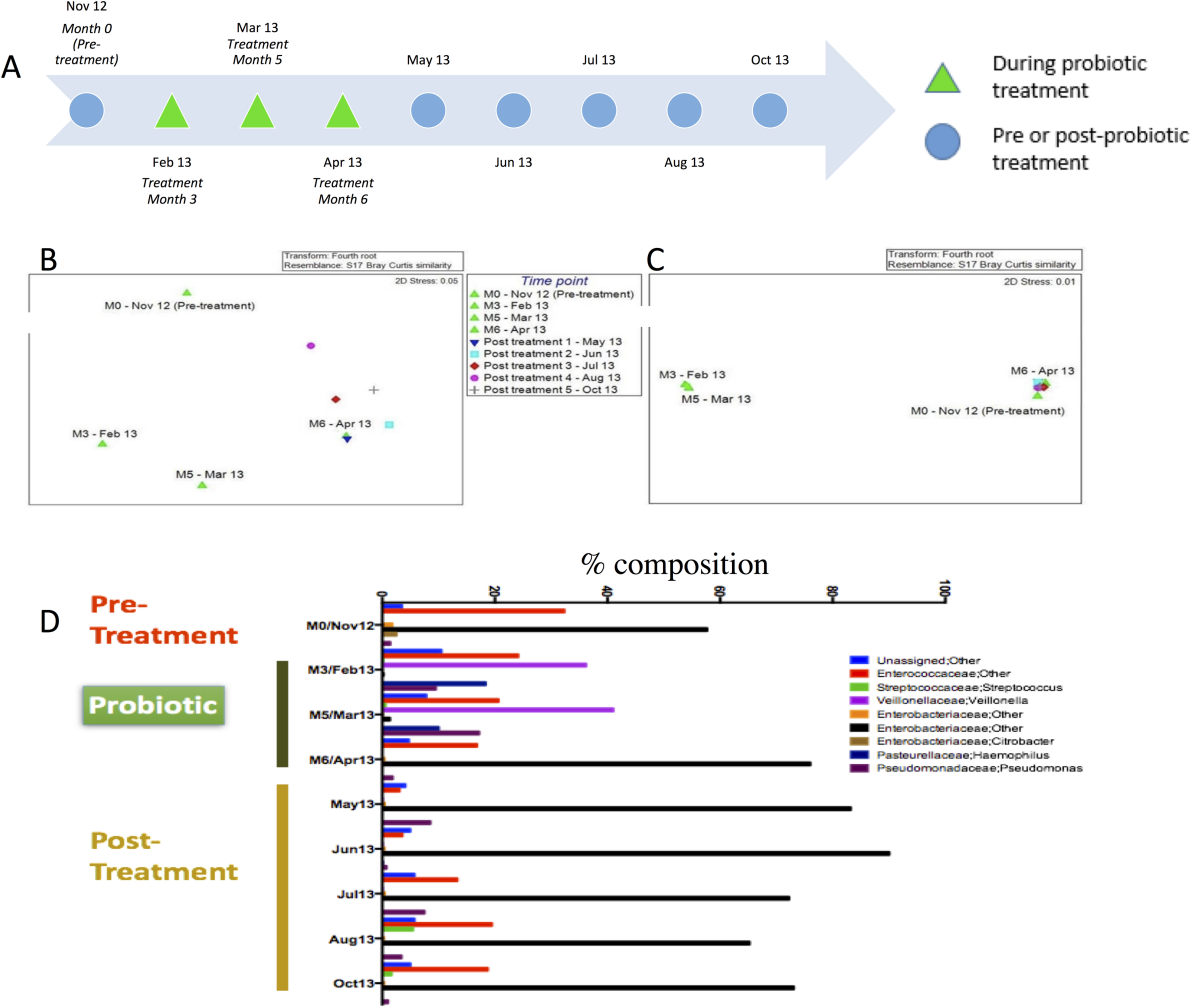
The microbial community composition from Patient 3 during and after probiotic treatment. (A) Samples and time line for Patient 3. MDS plot of the microbial flora from Patient 2 during and after probiotic treatment based on T-RFLP (B) and Illumina sequencing data (C). The four green triangles represent the samples collected during the probiotic interventional period between November 2012 and April 2013, with November being the baseline time point examined. All other samples were collected at five post-treatment time points between May and October 2013. D. Microbial flora composition at the genus level for Patient 3 during and after probiotic treatment based on Illumina sequencing data. The samples collected from November 2012 to April 2013 correspond to Month 0 (Pre-treatment), and Months 3, 5 and 6 of probiotic intervention. The post-treatment samples were collected at five time points between May 2013 and October 2013. Only OTUs representing at least 1% of the total community are shown. OTUs that were not resolved at the genus level are referred to by their lowest identified phylogenetic classification.

For Patient 3, there were four samples collected during the probiotic interventional period, at Month 0 (Pre-treatment) and at Months 3, 5 and 6 of treatment (Fig 6). The probiotic interventional period was between November 2012 and April 2013 (green triangles), and the patient was sampled at five time points after ceasing treatment (non-green triangles), from May to October 2013. MDS plots (Fig 6) showed temporal differences between probiotic treatment (green triangles) and post-treatment samples (non-green triangles). Comparison of the communities by T-RFLP and by sequence analysis (Fig 6A and 6B) indicated that there was a difference in the communities during probiotic treatment versus no treatment, although that difference was not statistically significant (p = 0.099 for T-RFLP data and p = 0.081 for the sequencing data, PERMANOVA). The difference between communities was especially clear for the sequence based data where the communities segregated into two distinct groups based on treatment (Months 3 and 5 samples on the left in Fig 6B) or no treatment (samples on the right, including the Pre-treatment sample in Fig 6B). Surprisingly, the sample collected at Month 6 (green triangle, Apr 13) of probiotic treatment appeared to be similar to the no treatment samples (non-green triangles) (Fig 6A and 6B).

To further understand the differences in communities between the samples collected at different time points, the community sequence data was examined based on changes in OTUs at the genus level for each time point. Eight different bacterial OTUs were identified at the genus level (Fig 6C) over the probiotic treatment period (November 2012 to April 2013) and the follow-up period (May 2013 to October 2013). The dominant taxon during probiotic treatment (at Months 3 and 5) was *Veillonella*, representing an average of 39% of the total community (Fig 6C). In contrast, before and after probiotic treatment, *Veillonella* represented an average of 0.5% of the microbial community. During non-treatment periods, the community was instead dominated by taxa of the family *Enterobacteriaceae* (OTU 225) at an average of approximately 74% (Fig 6C). During probiotic treatment, *Enterobacteriaceae* (OTU 225) declined sharply, representing 0.5% of the community. Another change during probiotic treatment was *Haemophilus*, which increased dramatically at Months 3 and 5 (average representation of 14%) compared to time points of no-treatment (not detectable, Fig 6C). *Pseudomonas* was also more abundant during Months 3 and 5 of probiotic treatment (13%), compared to no-treatment time points (4%, Fig 6C). Interestingly, the community at Month 6 of treatment (Mar 2013) was more similar to that of the non-treatment time points than it was to the samples collected during treatment. *Enterococcaceae* (OTU 101) was present at all time points, at an average of 17% of the total community.

## Discussion

This study compared urinary catheter communities of three male patients over time, with the aims of identifying the constituent microbial community of the neurogenic bladder and exploring inter-individual differences in microbiota as well as identifying intra-individual changes relating to probiotic therapy and clinically significant UTI episodes. T-RFLP and metacommunity amplicon sequencing were used to profile microbial communities. The key findings of the study were that the microbiota of catheters from patients with neurogenic bladders appeared to be diverse and patient specific, suggesting that there was no consensus microbiome that can be used to define a healthy or diseased state, based on the limited number of patients tested here. This is consistent with previous studies of urine from healthy males in which a universal microbial community profile could not be identified by sequencing [24,29,46,47]. Sequencing of urine from individuals with neurogenic bladders [21,48] and non-neurogenic bladders [49] also revealed patient-to-patient variation in urinary tract communities. This trend has also been reported for gut communities [50,51]. However, in our study, the composition of the microbiota all three patients also changed as a result of disturbances, including the intake of probiotics or the onset of symptomatic UTI. This preliminary data suggests that changes in an individual’s catheter associated microbiome has the potential to be used to monitor health status.

In two of three patients, probiotic therapy correlated with a change in the individual’s catheter associated microbial community. In Patients 1 and 3, the community observed during probiotic treatment clustered separately from their post-treatment microbiota. However, there was no difference observed between probiotic-associated and non-probiotic microorganisms for Patient 2. It is important to note that there was only one sample from the probiotic treatment period for Patient 2, making the study underpowered for testing the effect of the probiotic treatment. For Patients 1 and 3, while there was a distinct impact of probiotic treatment on the microbial community, the changes were transient. When treatment ceased, the patient’s microbial community profile shifted away from the probiotic-associated profile to the pre-probiotic profile for Patients 1 and 3. In Patient 3, this shift back to a pre-probiotic profile was seen as early as one month after stopping treatment. It is also possible that these differences between Patients 1 and 3 and Patient 2 is related to the site of catheterization and this should be controlled for in future studies, with increased patient numbers. Closer scrutiny of Patient 3’s samples also revealed that at Month 6 of probiotic treatment, the microbial community resembled the post-treatment community rather than probiotic microbiota. This observation, coupled with data from follow-up contact, suggested that Patient 3 was non-compliant with the treatment at this time point. Thus, the observation that the community changes depending on whether a patient was taking oral probiotic or not, suggests that it may be possible to monitor compliance with probiotic therapy by molecular profiling of urinary tract flora in this patient group by using the catheter associated micoorganisms as a proxy.

The change in the composition of microorganisms observed after ingesting probiotics is consistent with previous literature showing that orally administered *L*. *rhamnosus* GR1 and *L*. *fermentum* RC14 induce changes in vaginal flora [52]. In particular, treatment with GR1-RC14 resulted in increased levels of *Lactobacilli* in vaginal flora, and a decreased abundance of yeast species when examined by culturing of vaginal swabs [52]. Another study focusing the faecal community of infants over time by T-RFLP demonstrated that the faecal microbiomes were different between infants and that community composition varied during breastfeeding compared to when the infant was weaned. *Enterobacteriaceae* were particularly dominant during breastfeeding but declined after weaning [20]. One additional implication from our study is that orally delivered dietary treatments such as probiotics do influence the catheter-associated microbiota within the urinary tract of spinal cord patients.

Our data also suggest that orally delivered probiotic strains can alter the microbiota of the urogenitral tract in a relatively short time frame. Further, once treatment was stopped, the patient’s catheter community returned to the pre-treatment community composition, demonstrating that the bacterial composition of this niche is relatively stable and resilient. This observation is consistent with previous studies suggesting that probiotic strains do not colonise the bladder over the long-term [32,53,54]. The lack of a long term impact of probiotic treatment has also been reported for the intestinal community [55]. For example, within two weeks following oral administration of probiotic *Bifidobacterium animalis*, that species was almost undetectable in the faeces [56]. Thus, our study supports evidence that probiotics have a transitory effect on the composition of urogenital flora, and that long-term colonisation of the host with probiotic strains is challenging. It further demonstrates that sequencing or fingerprinting methods can be used to track treatment compliance as well as the effect of such treatments on the microbial communities of individual patients. As noted above, the limited number of patients reported here represents preliminary data that would support more detailed investigation of these questions in the future.

Patient 2 experienced one clinically significant UTI that was correlated with a concomitant change the microbial composition on the catheter just prior to the UTI. After antibiotic treatment and resolution of the UTI (October), the community shifted back to a non-infected profile and remained stable as long as the patient was asymptomatic (October to February). This indicates that the individual's microbial community may change or increase in diversity prior to UTI and is consistent with reports of microbial changes in the urinary tract prior to symptomatic UTI [57]. While it was determined by culturing that the patient was colonized with a multi-resistant strain of *E*. *coli*, although this organism was not detected in the sequencing data. This may be a due to the limitation of short read amplicon sequencing to resolve organisms at the species level or it could also reflect that, while *E*. *coli* was present, it was not the dominant organism associated with the catheter or was below detection limits. None the less, the study highlights the potential to use molecular approaches to track therapeutic compliance and may be predictive of infection, based on those changes to an individual’s catheter associated microbial community.

For each sample examined in this study, we observed concordance between the microbial community differences identified by T-RFLP and sequencing. Both methods detected a significant difference in communities between individuals as tested by PERMANOVA and the T-RFLP and Illimuna sequencing produced similar spatial separation of samples by patient and by time point. While T-RFLP has been widely used in microbial ecology (Mills et al., 2003), it has been used less extensively in human medicine. However, the work presented here shows that T-RFLP is sensitive enough to monitor changes in urinary tract flora. Compared to sequencing approaches, T-RFLP is relatively inexpensive, rapid to perform and can be simpler to interpret and hence may be suitable approach to monitor patients that are chronically catheterized, such as neurogenic patients. Thus, either T-RFLP or amplicon sequencing could be routinely performed on catheters upon their routine change. This would allow for the pattern of the community to be tracked over time and changes in that pattern could potentially indicate a disturbance in the community, e.g. invasion by a potential pathogen. This information could be used to as a management tool by clinicians to determine whether to change catheters more frequently or pre-emptive antibiotic therapy.

In summary, the data presented here highlight that each of the three patients were colonized by a community that was unique to the individual, and few organisms were shared across the three patients. Whilst the study population was small, the results from this longitudinal study of neurogenic patients undergoing probiotic therapy suggest that the catheter associated community was dynamic, where significant changes in the community composition were observed following the introduction of probiotic strains and changes in UTI status. Further, the results showed that the individual's microbial community was significantly different on and off probiotic treatment. The effect of probiotics on the urinary tract flora was transient, supporting evidence that long-term colonisation of the host with probiotic strains is difficult to establish. Thus, to realise the potential benefits of oral probiotics in the long term, it may require continuous administration of the treatment. While there was some evidence of change in the microbial composition prior to symptomatic UTI, further studies are required with a larger cohort of participants, and sampling of the urinary microbiome pre-, during and post-infection. This will be useful in determining the changes in microbiome structure that predict UTI. Finally, the study demonstrates the potential of molecular methods, i.e. T-RFLP or metacommunity sequencing, to monitor individual patient health and suggests that these methods could represent an important tool in the management of UTIs, especially for chronically catheterized patients.
